# Electronic cigarette use behaviors and motivations among smokers and non-smokers

**DOI:** 10.1186/s12889-017-4671-3

**Published:** 2017-09-08

**Authors:** Thomas E. Sussan, Fatima G. Shahzad, Eefa Tabassum, Joanna E. Cohen, Robert A. Wise, Michael J. Blaha, Janet T. Holbrook, Shyam Biswal

**Affiliations:** 1 Department of Environmental Health and Engineering, Johns Hopkins Bloomberg School of Public Health, 615 N. Wolfe Street, Baltimore, MD USA; 2 Department of Health, Behavior and Society, Institute for Global Tobacco Control, Johns Hopkins Bloomberg School of Public Health, Baltimore, MD USA; 3 Department of Medicine, Division of Pulmonary and Critical Care Medicine, Johns Hopkins School of Medicine, Baltimore, MD USA; 4 Department of Medicine, Ciccarone Center for the Prevention of Heart Disease, Johns Hopkins School of Medicine, Baltimore, MD USA; 5 Department of Epidemiology, Center for Clinical Trials and Evidence Synthesis, Johns Hopkins Bloomberg School of Public Health, Baltimore, MD USA; 6 Present Address: United States Army Public Health Center, Toxicology Directorate, Aberdeen Proving Ground, Aberdeen, MD USA

**Keywords:** Electronic cigarettes, Vapor, Preferences, Survey, Smokers, Baltimore, Maryland

## Abstract

**Background:**

The use of electronic cigarettes (EC) has risen exponentially over the past decade, including among never smokers, and ECs are now the most popular tobacco product among teenagers in the US. While, EC manufacturers utilize numerous marketing strategies to target both smokers and non-smokers, it is unclear how perceptions and behaviors differ between these two groups.

**Methods:**

We conducted a survey of 320 adults either via online surveys or in Baltimore vape shops to determine demographics, behaviors, perceptions, and motivations underlying use of ECs.

**Results:**

Our survey respondents were predominantly young, Caucasian males, 74% of whom identified themselves as former smokers, while 20% identified as current smokers and 6% were never smokers. Former smokers reported a longer history of EC use and higher nicotine concentrations than current smokers. For former and current smokers, the primary motivation for EC use was assistance to quit smoking, and nearly half indicated that they plan to reduce their nicotine concentration and eventually quit using ECs. Among former smokers, self-reports on use and measures of dependence were consistent with nicotine replacement as their primary motivation. The majority of former and current smokers also reported that their respiratory health had improved as a result of EC use, although this effect was stronger for former smokers. Never smokers reported less frequent EC use and dependence compared to former and current smokers. Their motivations for use were more commonly for enjoyment and popularity, and they displayed a reduced desire to eventually quit using ECs.

**Conclusions:**

These responses provide insight into the underlying thoughts and behaviors of smoking and non-smoking EC users and also suggest that never smoking EC users are an emerging demographic with different motivations and perceptions than those of current and former smokers.

## Background

Electronic cigarettes (ECs), which are battery-powered devices that deliver aerosolized nicotine and other constituents [1], have grown exponentially in popularity since first coming to the US market in 2006, including among adolescents and never smokers. In 2015, the US EC market reached $3.5 billion and is projected to exceed $20 billion by 2025 [2, 3]. Recent Centers for Disease Control and Prevention (CDC) reports showed that ECs have surpassed tobacco cigarettes as the most popular “tobacco” product among teenagers, with more than 3 million teenagers currently using ECs [4, 5]. According to the CDC, EC use increased from 1.5% in 2011 to 16.0% in 2015 among high school students and from 0.6% to 5.3% among middle school students. The use of ECs has also increased dramatically among young adults aged 18–24 years, with 21.6% of these individuals reported to have tried ECs and 5.1% who are current users [6].

Use of ECs remains a polarizing issue, including concerns related to: (1) whether they serve as an effective aid for smoking cessation versus whether they actually renormalize smoking and will reverse decades of measures that have reduced smoking rates; (2) whether or not never-smokers or former-smokers will be attracted to ECs and become regular users; and (3) whether or not there will be acute or long-term health consequences associated with use and whether these health effects must always be contextualized with cigarette smoking. These diverse questions will require a broad array of future studies, but user behaviors, perceptions, and motivations underlie all of these issues.

There are many factors that may influence the popularity of ECs among both smokers and non-smokers, including the wide assortment of flavors, glamorization by celebrities, perceptions that ECs are harmless or substantially less harmful than tobacco cigarettes and advertising campaigns that are increasingly directed toward young adults and non-smokers [7–10]. Previous surveys have determined that motivations for use of ECs are focused on cessation of smoking [11, 12], while other studies demonstrate that use is largely recreational and centered on enjoyment [13]. Thus, motivations are likely to depend on many factors, including demographics and smoking status.

EC companies have invested heavily in market development, advertising, and innovation [8, 14]. In May 2016, the Food and Drug Administration (FDA) finalized a rule to extend its regulatory authority over electronic cigarettes [15]. At present, this rule restricts sales of ECs to minors, and in the near future each product will require FDA pre-market review before it can be sold. However, the FDA ruling did not restrict television or radio advertising of ECs. The manner in which these products are marketed and perceived is likely to impact their use and their net effect on public health.

The goal of our current study was to investigate behaviors, perceptions, and motivations underlying the use of ECs in adults. We hypothesized that perceptions and motivations would differ based on smoking status. The information provided in this study will enhance understanding of the underlying behaviors of EC users.

## Methods

The survey was conducted in three Baltimore area stores that sell EC/vaping products (referred to as vape shops) or online from December 2014 to July 2015. Oral consent was provided by both the store owner/manager and survey participant. Shops were chosen based on convenience and permission from the store owner. Surveys, which contained 43 questions, were self-administered by completing a paper form inside the store (*n* = 15) or online (*n* = 305), depending on the preference of the participant. Surveys with a majority of missing data or otherwise unusable responses were excluded (11 of 331 were excluded). Residential zip codes for 207 of the respondents were within Maryland, while 107 responses were outside of Maryland and 6 were missing. This study was approved by the Institutional Review Board of the Johns Hopkins University. Oral consent was deemed sufficient since this was a voluntary survey that collected no identifying or personal information.

Based on responses to multiple questions, respondents were classified into one of three groups depending on their reported history of cigarette smoking: never smokers, former smokers, and current smokers. Never smokers were individuals who smoked fewer than 100 cigarettes in their lifetime. Former smokers were individuals who identified themselves as quitters and reported not smoking a cigarette in the past 30 days. Current smokers were individuals who reported having smoked at least 100 cigarettes in their lifetime and at least 1 in the past 30 days. Because there were relatively few never smokers (*N* = 19, 6% of study respondents), we focused our statistical analysis on the comparisons between former and current smokers. However, we did include those responses from never smokers to observe how they differed from former or current smokers. Demographic characteristics and responses to the survey were compared between current and former smokers by Chi-square tests and Fisher’s Exact Test if a cell size was 5 or less. Concordance between duration of smoking cessation and duration of EC use among former smokers was examined with an agreement chart [16].

## Results

### Survey demographics

We received 331 survey responses, 320 with useable data. Over three-quarters of respondents were male, 89% were white, and over half were younger than 35 years of age (Table 1). 74% of respondents were former smokers, 20% were current smokers and 6% were never-smokers. Current smokers were younger than former smokers (*p* = 0.05). Never smokers were younger than both former and current smokers. Smoking status was also related to educational attainment, marital status and employment status, which was likely due to the age differences among the groups.

**Table 1:** Survey respondent demographics by smoking status

Characteristic	Total N (%)	Former smoker N (%)	Current smoker N (%)	*p*-value*	Never smoker N (%)
Respondents^a^	320 (100)	237 (74)	64 (20)		19 (6)
Age					
18–24 years old	57 (18)	31 (13)	13 (20)	0.05	13 (68)
25–34 years	129 (40)	94 (40)	31 (48)		4 (21)
35 years or older	134 (42)	112 (47)	19 (21)		2 (11)
Gender					
Male	248 (78)	182 (77)	48 (75)	0.76	18 (95)
Race					
Caucasian	284 (89)	214 (91)	55 (86)	0.27	15 (79)
Other	35 (11)	22 (9)	9 (14)		4 (21)
Education					
4 years college +	102 (32)	78 (33)	20 (31)	0.07	4 (21)
College 1 year to 3 years	137 (43)	106 (45)	21 (33)		10 (53)
Grade 12 or less	81 (25)	53 (22)	23 (36)		5 (26)
Marital status					
Unmarried couple	68 (21)	46 (19)	17 (27)	0.02	5 (26)
Married	113 (35)	97 (41)	13 (20)		3 (16)
Never married	110 (34)	72 (30)	27 (42)		11 (58)
Was married	29 (9)	22 (9)	7 (11)		0 (0)
Employment status					
Student	23 (7)	8 (3)	8 (13)	0.031	7 (37)
Employed	210 (66)	161 (68)	42 (66)		7 (37)
Not employed	40 (13)	29 (12)	7 (11)		4 (21)
Self-employed	47 (15)	39 (16)	7 (11)		1 (5)

*p-values from Chi-square tests for comparisons between current and former smokers

^a^This is percent of total respondents

### Tobacco use

The smoking history of former or current cigarette smokers was similar in that both groups reported starting tobacco use before age 18 years (Table 2). Former smokers reported a history of higher daily tobacco cigarette consumption than that reported by current smokers (*p* < 0.01).

**Table 2:** Tobacco cigarette and EC use characteristics

	Former smoker	Current smoker		Never smoker
	*N* = 237 N (%)	*N* = 64 N (%)	*p*-value*	*N* = 19 N (%)
Tobacco cigarette use				
Age started smoking				
< 18 years	153 (65)	46 (73)	0.15	N/A
18 or older	84 (35)	17 (27)		
Daily cigarette consumption^a^				
0–10 cigarettes	30 (13)	28 (47)	<.01	N/A
11–20 cigarettes	98 (43)	20 (33)		
More than 20 cigarettes	98 (43)	12 (20)		
EC use				
Length of EC use				
About 6 months or less	36 (15)	17 (27)	0.04	6 (32)
About 1 year	47 (20)	17 (27)		9 (47)
Between 1 and 2 years	83 (35)	19 (30)		3 (16)
2 years or more	71 (30)	11 (17)		1 (5)
EC use per day				
1–3 times per day	12 (5)	2 (3)	0.52	4 (21)
4 or more times per day	219 (95)	62 (97)		15 (79)
Use different nicotine concentrations				
Yes	36 (15)	8 (13)	0.59	1 (5)
Primary nicotine concentration (mg/ml)				
Missing	44 (19)	24 (38)	0.04	7 (37)
Low <6	73 (31)	18 (28)		9 (47)
Medium 6–15	90 (38)	18 (28)		2 (11)
High >15	30 (13)	4 (6)		1 (5)
Primary motive for using ECs				
Aid to quit smoking	100 (42)	25 (39)	0.12	NA
Cheaper than tobacco cigarettes	8 (3)	6 (9)		0 (0)
Healthier than tobacco cigarettes	89 (38)	21 (33)		1 (5)
Enjoyment	30 (13)	6 (9)		13 (68)
Other	10 (4)	6 (9)		5 (26)
Plan to reduce the nicotine in ECs				
Yes	184 (78)	53 (83)	0.66	6 (31)
Time from wakening to first EC				
Less than 30 min	143 (62)	39 (64)	0.64	3 (16)
Plan to quit using ECs				
Yes	94 (40)	29 (45)	0.72	4 (21)

Abbreviations: *EC* = Electronic cigarette, *N* = Total number, *NA* = not applicable

*p-values from Chi-square or Fisher Exact tests for comparisons between current and former smokers

^a^For former smokers, daily cigarette consumption is for use prior to smoking cessation

### Electronic cigarette use

Regardless of tobacco smoking status, most users reported using ECs at least 4 times per day (Table 2). Current smokers were more recent initiators of EC use compared to former smokers (*p* = 0.04), and also reported lower nicotine concentrations than former smokers (*p* = 0.04), although nicotine concentration had high non-response rates, which was at least partially due to a lack of knowledge of the concentration used. The majority of both groups, 62 to 64%, reported using an e-cigarette within 30 min of wakening, which was much higher than never smokers (16%). Nearly half of current and former smokers reported intentions to quit EC use eventually, and most intended to reduce nicotine concentration. Most never smokers reported using ECs one year or less (79%) and reported using lower nicotine concentrations than current or former smokers. There were no specific EC flavors that emerged as substantially more popular than others, as indicated by the 165 unique responses reported as their most preferred flavor.

**Fig. 1:**
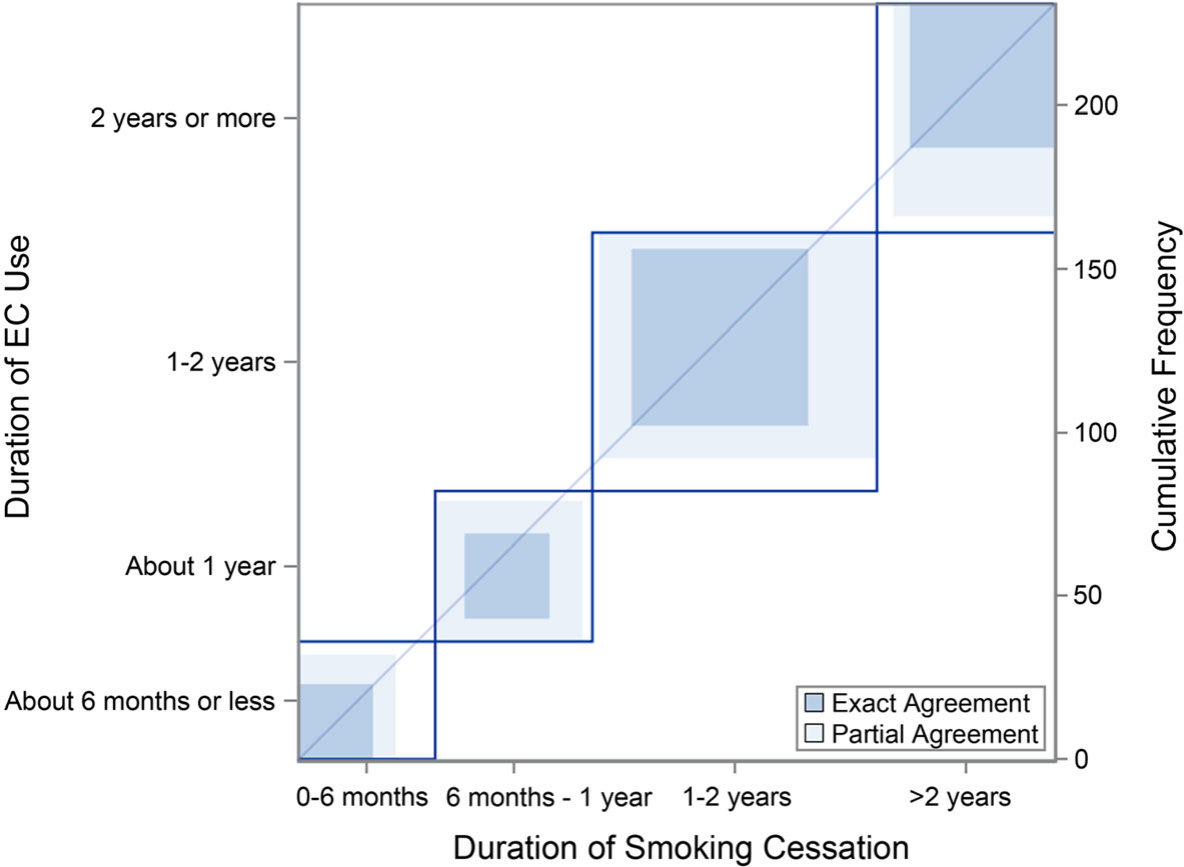
Agreement Chart for the duration of EC use and duration of smoking cessation among former smokers. The size of the rectangle at each time point reflects the marginal totals for each variable. The area of exact agreement is the dark square, which represents the number of individuals who reported the same duration for EC use and smoking cessation. The lighter rectangle represents the number of individuals who reported similar (within one category) durations. The white square area represents area of disagreement

The motivations for EC use did not significantly differ between former and current smokers. The most frequently cited primary motivation for initiation of EC use among both former and current smokers was to quit smoking tobacco (42% and 39%, respectively), followed by the belief that ECs were healthier than tobacco cigarettes (38% and 33%, respectively). Less commonly cited primary motivations included that ECs were cheaper than cigarettes and enjoyable (≤13%). Most never smokers (68%) said enjoyment was their primary motivation for use. Other primary motivations for never smokers included stress reduction and popularity. Health considerations were rarely (5%) a motivating factor for non-smokers. The relative lack of concern about health considerations may explain why only 21% of never smokers reported that they planned to quit EC use as compared to 40% of former smokers and 45% of current smokers. Since most former smokers reported that they started using ECs to help them stop smoking tobacco cigarettes, we evaluated the agreement between the initiation of EC use and duration of smoking cessation (Table 3 and Fig. 1). The agreement chart suggests that the initiation of ECs is closely tied with cessation of smoking.

**Table 3:** Duration of EC use and time since last cigarette among former smokers

	How long since last cigarette, N (% of total)				
How long used EC	0–6 months	6 months - 1 year	1–2 years	>2 years	Total
About 6 months or less	23	9	2	2	36 (16)
About 1 year	7	26	10	3	46 (20)
Between 1 and 2 years	10	10	54	5	79 (34)
2 years or more	2	3	21	44	70 (30)
Total	42 (18)	48 (21)	87 (38)	54 (23)	231

Missing data = 6

### Perceived health changes

Although relatively fewer former smokers reported persistent respiratory symptoms like a cough or wheezing commonly associated with smoking (20–44%) than current smokers (45–62%), a greater percentage of former smokers reported improvements in persistent respiratory symptoms (78–90%) and change in health status after starting EC use than did current smokers (55–69%, Table 4).

**Table 4:** Perceived health changes

	Former N (%)	Current N (%)	*p*-value
Health changes after starting use of ECs			
Have not paid attention to this	3 (1)	8 (13)	<.01
No	8 (3)	9 (13)	
Yes	226 (95)	47 (73)	
Persistent symptoms before starting ECs			
Cough	63 (27)	40 (62)	<.01
Wheeze	48 (20)	34 (53)	<.01
Chest tightness	53 (22)	29 (45)	<.01
Sputum	82 (35)	30 (47)	0.07
Sinus	104 (44)	35 (55)	0.12
Symptoms improved after start using ECs^a^			
Cough	57 (90)	24 (60)	<.01
Wheeze	43 (90)	22 (65)	<.01
Chest tightness	46 (87)	16 (55)	<.01
Sputum	64 (78)	16 (55)	0.01
Sinus	83 (81)	24 (69)	0.17

*P-value determined with Chi-square test or Fisher’s Exact Test if any response category had fewer than 5 respondents

^a^Limited to respondents reporting symptoms

## Discussion

Our survey is one of the first to report behaviors and preferences of EC users among respondents recruited from vape shops. Our survey respondents were predominantly young, Caucasian males, which is the most common demographic among current EC users [6]. Additionally, 6% of the respondents indicated that they were never smokers, although it is possible that the cross-sectional design of our study could lead to an over-representation of this group since our study would have failed to capture those individuals who had quit nicotine entirely after transient use of ECs. Furthermore, never smokers who use ECs may be more likely to visit vape shops than former or current smokers who are accustomed to purchasing cigarettes in other types of stores. Nonetheless, the prevalence of never smokers identified here is consistent with several studies from the US suggesting that an increasing number of EC users are never-smokers [6, 17–19]; while it conflicts with a highly publicized report by Public Health England indicating that a negligible number of never smokers regularly use ECs [20]. These differing conclusions likely contribute to the contrasting policy guidelines between the US and UK and may reflect localized risks or priorities (i.e., policies that focus on non-smokers and children versus smokers). These policy makers have access to the same data, which highlights the importance of history and values in determining policy goals and interventions. Our survey supports that never-smoking EC users are in the minority, but with approximately 7 million adults in the US who are current EC users (3.5% of US adults) [19], this represents an alarming number of predominantly younger individuals who are using ECs. While smokers may experience a potential benefit from EC use, never smokers who use ECs likely receive no benefit. However, it could be argued that never smokers who use ECs may have otherwise turned to a more dangerous alternative if not for ECs. Thus, further evidence is needed to inform the most appropriate regulatory measures.

Among our survey respondents, the never-smoking group was younger than former and current smokers. This is consistent with results from the 2015 CDC’s National Health Interview Survey, which showed that while 11.4% of adult EC users were never smokers, the percentage was highest among those aged 18–24 years (40%) and lowest among those aged ≥45 years (1.3%) [19]. Thus, the prevalence of never-smoking EC users may depend on the age of the surveyed population, and these data may suggest an alarming trend in which increasing numbers of young adults who have never smoked tobacco are becoming regular users of ECs. Furthermore, while advertising of tobacco products is aggressively limited, EC advertising is not. Spending on EC advertising in 2014 was $115 million, and 69% of middle and high school students (18 million youths) were exposed to EC advertisements in retail stores, on the Internet, in magazines/newspapers, or on TV/movies [14]. Advertising can be quite influential to teens and may further increase the prevalence of ECs among youth. Given the unknown health risks of ECs and no possibility of harm reduction among non-smokers, regulators may consider more rigorous limitations on the sale and advertising of ECs.

Our comparison of cigarette use between former and current smoking EC users revealed that current smokers report smoking fewer cigarettes per day than former smokers did prior to quitting, while former smokers reported using higher nicotine concentrations and using ECs longer than current smokers. These differences suggest that current smokers were at an earlier stage in their transition from tobacco cigarettes to ECs and were titrating their cigarette use and supplementing with ECs. For both groups, the primary motivation for initiating EC use was as an aid to quit smoking, and nearly half of current and former smokers indicated that they plan to reduce their nicotine concentration and eventually quit using ECs. While many smokers use ECs as a smoking cessation aid and some studies support the notion that ECs reduce cravings and cigarette consumption [21, 22], the effectiveness of ECs as a smoking cessation aid has been questioned [23]. While our study did not directly address the efficacy of ECs as a smoking cessation aid, most current and former smokers reported that they had either substantially reduced or completely stopped using cigarettes as a result of EC use.

Furthermore, the agreement observed between duration of smoking cessation and duration of EC use among former smokers suggests that users quickly transitioned to sole use of ECs. However, the success rate of complete cessation of nicotine use after transitioning from cigarettes to ECs is unknown. While the most commonly reported primary motivation for using ECs among both former and current smokers was as an aid to quit smoking, it is unclear why the current smokers continue to smoke after initiation of ECs. It is possible that current smokers may have previously reduced their cigarette consumption via conventional means prior to EC initiation, resulting in diminished effectiveness of ECs as a smoking cessation aid.

Among current and former smokers, the second most common motivation for use of ECs was the belief that ECs were a healthier alternative to smoking. Consistent with this perception, the majority of both current and former smokers reported that their respiratory health had improved as a result of the initiation of ECs. However, former smokers were more likely to report improved respiratory symptoms, suggesting that switching to ECs completely has greater benefit than dual use. Interestingly, former smokers were less likely to report respiratory symptoms than current smokers prior to EC initiation, despite high cigarette consumption. The explanation for this is unclear, although it is possible that this difference is due to recall bias and the longer length of time since smoking. It is also possible that former smokers were more likely to quit due to concerns about hypothetical health risks; while current smokers persisted in smoking even after development of respiratory symptoms and were either not as concerned about health risks or were unable or unwilling to completely quit smoking.

Several studies in humans and animal models suggest that use of ECs has detrimental effects on pulmonary health [24–26], although these effects are often reported to be milder than for cigarettes [26–28]. Our results support that EC use may reduce harm based on self-reported respiratory symptoms, but our results are likely biased by respondents’ expectations, and more definitive and longitudinal studies are needed. EC use may have inherent health risks not associated with tobacco smoking, or for certain health outcomes, it is possible that ECs may pose more significant harm than smoking. For example, several studies demonstrate that EC use leads to immunosuppression [29, 30], and this immunosuppressive effect may be greater for ECs than for cigarette smoke [31]. The long-term health effects of chronic EC use are not known, although the effects on respiratory symptoms reported by regular EC users in our survey were generally positive compared to cigarette smoke. However, considering that 6% of our study population were never smokers, it appears to be inappropriate to only consider the health effects of ECs in the context of reduction or elimination of cigarette use, especially since patterns of use in teenagers suggest that the number of new EC users who have never smoked cigarettes is likely to continue to rise [32].

Never smokers may be less reliant on EC use, as only 16% of non-smokers used their first EC within 30 min of awakening compared to over 60% of former and current smokers, indicating less dependence on nicotine. Their primary motivations for use were enjoyment and popularity, which may explain their lower expectation to eventually discontinue EC use. These findings suggest that the daily use of ECs among never smokers is an emerging public health concern. Future regulations may prohibit marketing strategies aimed at non-smokers or teens, who are the likely targets of many current marketing strategies and receive no direct benefit from use. These marketing strategies include flavor options that appeal to younger people. In our survey, we asked individuals to identify their preferred EC flavor; however, we received 165 unique responses. This diversity of flavors may contribute to the efficacy of ECs as a smoking cessation aid by allowing many choices. The wide variety of EC flavors are appealing to both smokers and non-smokers [33]; however, restricting flavor options may reduce EC appeal to never smokers without substantially effecting efficacy of EC as a smoking cessation aid. Regulations should attempt to minimize initiation of EC use by never smokers; however it may not be appropriate to levy regulations to the point where ECs are completely rejected by smokers.

While this study identified key differences among never smokers, one important limitation of this study was the small sample size of the never smoking group. Future studies are needed to assess the motivations and perceptions among this key demographic. Additional limitations are that we recruited survey participants from vape shops, which may not represent the entire EC using population. These users may have greater interest in staying on the cutting edge of EC technology. Furthermore, our cross-sectional analysis of current vape shop customers did not capture individuals who successfully quit smoking via temporary use of ECs, and thus provides no information on the transition to no use. Lastly, two-thirds of our respondents resided in Maryland, and thus this survey may not reflect all regions of the US.

## Conclusions

This study elucidates that characteristics of EC users vary by smoking status and reveals striking differences in motivations and perceptions based on smoking status. These differences will aid in understanding factors that drive EC usage, and may be informative for regulatory and marketing purposes. Also, the results of this survey should inform the design of future studies. For example, since more than half of the EC users were young adults (18–34 years) who are unlikely to present with overt disease, longitudinal follow-up of EC users should focus on sub-clinical disease endpoints and biomarkers. Furthermore, studies must be prepared to follow a mobile population over time. Future studies with larger sample sizes and prospective observation will help us to understand how perceptions and behaviors continue to evolve and to determine the impact of the new FDA regulations.

## Abbreviations

CDC: Centers for Disease Control and Prevention
EC: electronic cigarette
FDA: Food and Drug Administration
