# Correction to: electronic cigarette use behaviors and motivations among smokers and non-smokers

**DOI:** 10.1186/s12889-018-5048-y

**Published:** 2018-01-24

**Authors:** Thomas E. Sussan, Fatima G. Shahzad, Eefa Tabassum, Joanna E. Cohen, Robert A. Wise, Michael J. Blaha, Janet T. Holbrook, Shyam Biswal

**Affiliations:** 1 Department of Environmental Health and Engineering, Johns Hopkins Bloomberg School of Public Health, 615 N. Wolfe Street, Baltimore, MD USA; 2 Department of Health, Behavior and Society, Institute for Global Tobacco Control, Johns Hopkins Bloomberg School of Public Health, Baltimore, MD USA; 3 Department of Medicine, Division of Pulmonary and Critical Care Medicine, Johns Hopkins School of Medicine, Baltimore, MD USA; 4 Department of Medicine, Ciccarone Center for the Prevention of Heart Disease, Johns Hopkins School of Medicine, Baltimore, MD USA; 5 Department of Epidemiology, Center for Clinical Trials and Evidence Synthesis, Johns Hopkins Bloomberg School of Public Health, Baltimore, MD USA; 6 Present Address: United States Army Public Health Center, Toxicology Directorate, Aberdeen Proving Ground, Aberdeen, MD USA

## Correction

After publication of the article [1], it has been brought to our attention that there is a funding acknowledgement missing. The authors would also like to include “Dr. Michael Joseph Blaha is funded by the American Heart Association Tobacco Regulatory Center, funding number: 1P50HL120163”.
